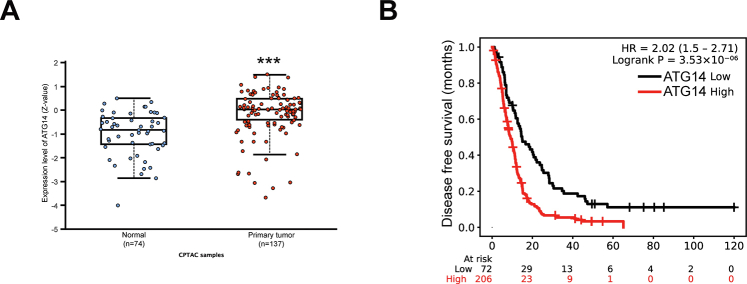# USP1 promotes pancreatic cancer progression and autophagy by deubiquitinating ATG14

**DOI:** 10.1016/j.jbc.2025.108190

**Published:** 2025-01-13

**Authors:** Leilei Li, Zhili Fan, Mengfei Liu, Hao Dong, Jing Li, Yu Li, Zan Song, Ying Liu, Zhicheng Zhang, Xinyu Gu, Tao Zhang

**Affiliations:** 1 Institute of Immunopharmaceutical Sciences, NMPA Key Laboratory for Technology Research and Evaluation of Drug Products, Key Laboratory of Chemical Biology, School of Pharmaceutical Sciences, Cheeloo College of Medicine, Shandong University, Jinan, Shandong, China; 2 State Key Laboratory for Chemistry and Molecular Engineering of Medicinal Resources, Guangxi Normal University, Guilin, Guangxi, China

**Keywords:** USP1, pancreatic ductal adenocarcinoma, autophagy, ATG14, cisplatin sensitivity

## Abstract

Pancreatic ductal adenocarcinoma (PDAC) is characterized by extremely poor prognosis, high mortality, and limited therapeutic strategy. Autophagy is hyperactivated in PDAC, and targeting autophagy is emerging as a promising therapeutic strategy. The dysfunction of deubiquitinase ubiquitin-specific peptidase 1 (USP1) results in tumorigenesis and chemotherapy resistance. However, little is known about how USP1 regulates autophagy and its mechanism in tumor progression and drug sensitivity in PDAC. In this study, we found USP1 elevated in pancreatic cancer and USP1 expression inversely correlated with overall survival. USP1 depletion inhibited cell proliferation, epithelial–mesenchymal transition, and migration in PDAC cells. Interestingly, USP1 knockdown or inhibition reduced autophagy initiation and autophagy flux. By screening of interacting protein using coimmunoprecipitation, we identified that USP1 interacted with ATG14 (autophagy-related gene 14) protein, acting as a core component in autophagy initiation. Furthermore, USP1 overexpression deubiquitinated and enhanced ATG14 protein stability by reduced binding ubiquitin levels, whereas USP1 inhibition promoted its proteasome-dependent degradation. Notably, USP1 depletion or a novel USP1 inhibitor I-138 dramatically delayed tumor growth in xenograft model. USP1 inhibitor synergistically enhanced the anticancer efficiency of cisplatin in PDAC cells. Collectively, our study identifies USP1 as the first deubiquitinase in the modulation of ATG14 deubiquitination and unveils a regulatory role for USP1 in autophagy and PDAC progression. Targeting USP1 using a selective inhibitor I-138 may provide an effective strategy for chemotherapy treatment and combating drug resistance in autophagy-activated pancreatic cancer.

Pancreatic ductal adenocarcinoma (PDAC) accounts for approximately 90% of pancreatic cancer, which is characterized by extremely poor prognosis and high mortality, and the average 5-year overall survival rate is only about 10% (1). Currently, the mainstay of conventional systemic therapy for advanced and metastatic PDAC patients is combination chemotherapy including FOLFIRINOX (5-fluorouracil, leucovorin, irinotecan, and oxaliplatin), gemcitabine plus nab-paclitaxel, and gemcitabine plus cisplatin (2, 3). However, the aforementioned combination chemotherapy has not offered a dramatically satisfying therapeutic efficacy with less than 1 year of overall survival (4, 5). Thus, there is an urgent need to unveil novel molecular characterization and yield potential therapeutic strategies for PDAC.

Autophagy is an evolutionarily conserved intracellular degradation process, which sustains cell metabolism and survival under starvation and stress conditions, and eliminates damaged proteins and organelles to maintain protein and organelle quality (6). Autophagy is hyperactivated and supports tumor growth in the progression of premalignant pancreatic intraductal neoplasia (PanIN) to pancreatic cancer, indicating that autophagy plays an important role in PDAC progression (7, 8). The inhibition of autophagy by chloroquine manifests robust tumor regression and enhanced sensitivity to first-line drugs, gemcitabine and oxaliplatin, in PDAC xenografts and genetic mouse models (9, 10), suggesting that targeting autophagy is remarkably effective in combating PDAC progression in preclinical studies. Unfortunately, the anticancer effect of autophagy inhibitor hydroxychloroquine remains frustrating in PDAC clinical trials (11), which is partially because of unknown complex mechanisms of autophagy and poor selectivity of autophagy inhibitor. Thus, identification of novel molecular mechanisms of autophagy and targeting core autophagy modulators using selective inhibitors may provide a reasonable clinical strategy in PDAC treatment.

Protein homeostasis regulated by ubiquitin enzymes and deubiquitinating enzymes (DUBs) is essential for sustaining biological activities (12). Dysregulation of DUBs is involved in various human disease, including cancer (13). It has been demonstrated that ubiquitin-specific peptidase 1 (USP1) is abnormally overexpressed in breast cancer, ovarian cancer, glioma, and osteosarcoma (14, 15). USP1 regulates DNA damage repair, cell cycle, and apoptosis, subsequently participating in tumor growth and metastasis, suggesting that USP1 may be a potential drug target (15, 16). In addition, USP1 induces the progression of PanIN stages to PDAC by modulating branched-chain amino acid metabolism (17). However, little is known about the role and mechanism of USP1 in modulating autophagy and chemoresistance of PDAC. Furthermore, whether targeting USP1 using a selective inhibitor represents a novel vulnerability in PDAC has yet to be determined.

In this study, we found that elevated USP1 expression is correlated with poor prognosis in PDAC. USP1 depletion inhibits cell proliferation, migration, and tumor growth *in vitro* and *in vivo*. Importantly, USP1 interacts with core autophagy protein ATG14 (autophagy-related gene 14), which deubiquitinates and increases its protein stability to facilitate autophagy initiation and autophagy flux. Moreover, pharmaceutical inhibition of USP1 significantly reduces tumor formation using a novel USP1 inhibitor I-138 in PDAC xenograft model. In addition, USP1 inhibition enhances sensitivity of PDAC cells to cisplatin and shows a synergistic antitumor effect. Overall, our findings identify the first deubiquitinase for ATG14 and uncover the exact role and mechanism of USP1 in autophagy and PDAC progression. Targeting USP1 provides a potential strategy for autophagy-activated pancreatic cancer.

## Results

### Upregulated USP1 expression is negatively correlated with overall survival

**Figure 1:**
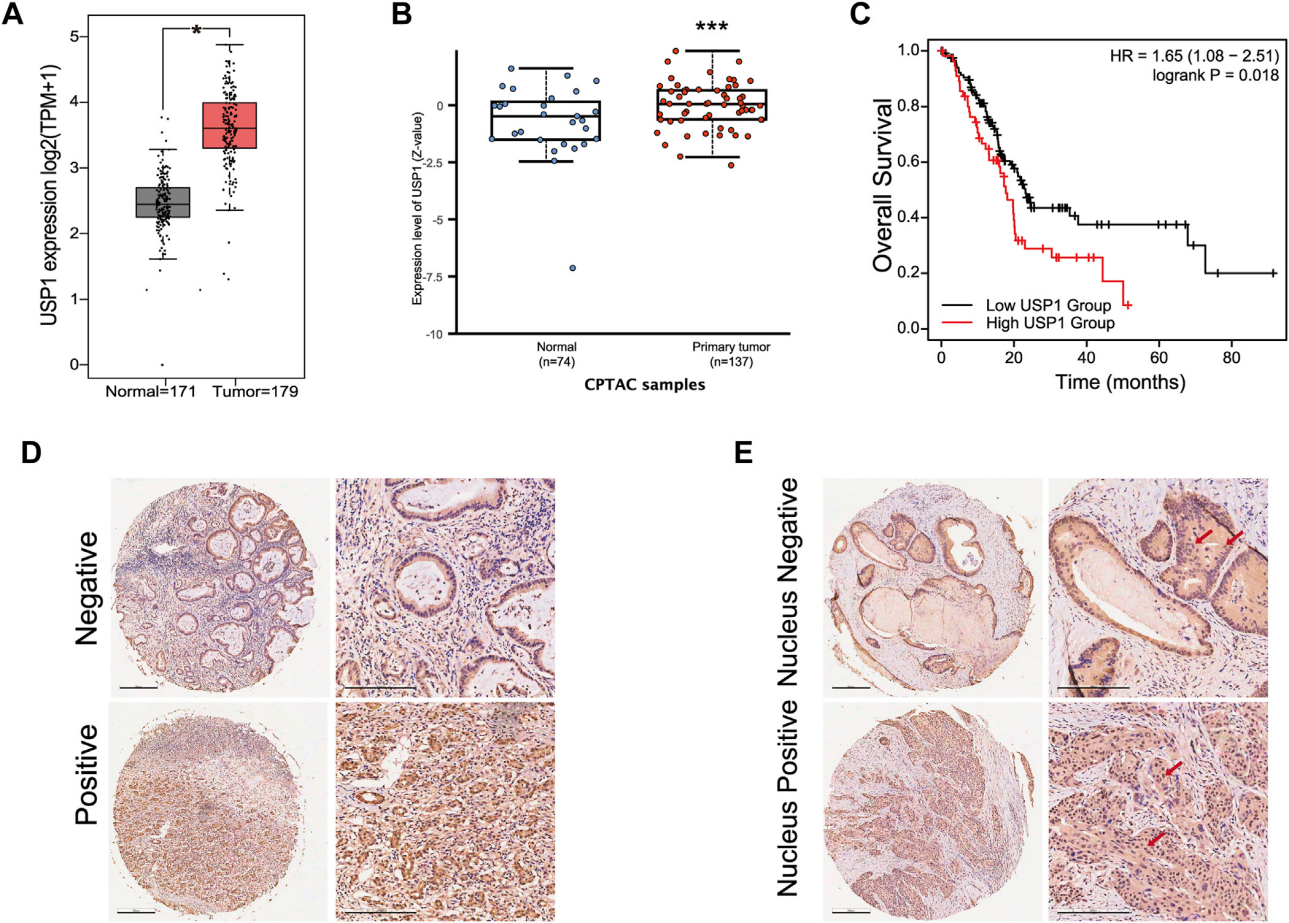
**High USP1 expression is associated with poor prognosis in PDAC.***A*, the mRNA level of USP1 in normal pancreatic tissues and PDAC tissues. *B*, the USP1 protein expression in normal pancreatic tissues and PDAC tissues. *C*, correlation between USP1 expression and overall survival. *D*, representative immunohistochemistry (IHC) images of negative and positive USP1 staining in PDAC microarray. *E*, representative IHC images of nucleus negative and positive USP1 staining in pancreatic duct tissues as shown with a *red arrow*. Bar represents 300 μm (*left panel*), 200 μm (*right panel*), compared with normal tissue, ∗*p* < 0.05, ∗∗*p* < 0.01, and ∗∗∗*p* < 0.001. PDAC, pancreatic ductal adenocarcinoma; USP1, ubiquitin-specific peptidase 1.

Although USP1 has been reported to participate in numerous cancer types, the specific role in pancreatic cancer remains largely unclear. To determine USP1 expression in normal and tumor tissue samples, we analyzed the mRNA and protein levels of USP1 from The Cancer Genome Atlas and Clinical Proteomic Tumor Analysis Consortium dataset using GEPIA and UALCAN. The results showed that the mRNA and protein expression of USP1 in PDAC tissues were significantly elevated compared with normal tissues (Fig. 1, *A* and *B*). Kaplan–Meier survival analysis demonstrated that high USP1 level exhibited a remarkably poor overall survival (Fig. 1*C*). Then, the immunohistochemistry (IHC) staining of USP1 was performed using PDAC tissue microarray. The positive USP1 staining was identified in 47 (82%) of 54 PDAC tissues (Fig. 1*D*). Interestingly, we observed that 31 (57%) of 54 PDAC tissues showed a positive nuclear USP1 staining (Fig. 1*E*). Taken together, USP1 may serve as an oncogene in PDAC.

### USP1 promotes cell proliferation and migration in PDAC cell lines

**Figure 2:**
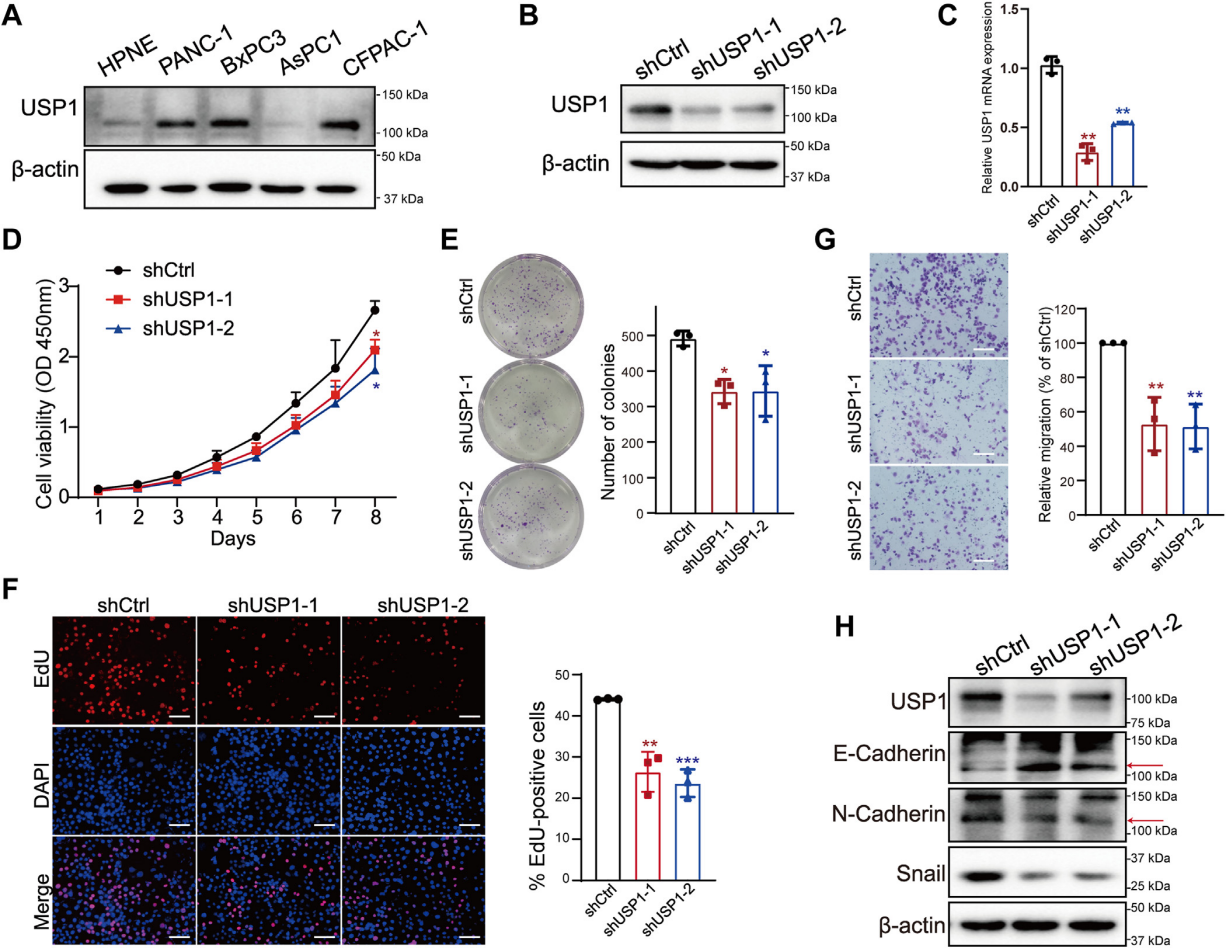
**USP1 promotes PANC-1 cell proliferation and migration.***A*, immunoblot analysis of USP1 expression in normal pancreatic cell line and PDAC cell lines. *B* and *C*, detection of USP1 knockdown efficiency at protein and mRNA levels in PANC-1 cells. *D*, PANC-1 cell proliferation ability of Control (shCtrl) and USP1 knockdown cell lines by CCK8. *E* and *F*, representative images and statistical graph of colony formation and EdU staining in shCtrl and USP1 knockdown cell lines. Bar represents 200 μm. *G*, effect of USP1 knockdown on cell migration ability in PANC-1 cells. Bar represents 200 μm. *H*, effect of USP1 knockdown on EMT-related proteins. The *red arrow* points to the target bands. Data are shown as mean ± SD from three independent experiments. Compared with shCtrl, one-way ANOVA, ∗*p* < 0.05, ∗∗*p* < 0.01, and ∗∗∗*p* < 0.001. CCK8, Cell Counting Kit-8; EdU, 5-ethynyl-2′-deoxyuridine; EMT, epithelial–mesenchymal transition; PDAC, pancreatic ductal adenocarcinoma; USP1, ubiquitin-specific peptidase 1.

We detected USP1 protein expression in different PDAC cell lines and found that USP1 expression was elevated in PDAC cells, including PANC-1, BxPC3, and CFPAC-1 compared with normal human pancreatic duct epithelial cell HPNE (Fig. 2*A*). To investigate the malignant behaviors of USP1, we successfully established USP1 knockdown cell lines in PANC-1 and BxPC3 (Figs. 2, *B* and *C* and S1, *A* and *B*). The Cell Counting Kit-8 (CCK8), colony formation, and 5-ethynyl-2′-deoxyuridine (EdU) assays showed that depletion of USP1 significantly inhibited cell proliferation *in vitro* (Figs. 2, *D*–*F* and S1, *C*–*E*). Furthermore, transwell assay revealed that USP1 knockdown markedly suppressed cell migration (Figs. 2*G* and S1*F*). Epithelial–mesenchymal transition (EMT) is closely associated with malignant properties of tumor cells and metastasis (18). In EMT process, epithelial cells lose their properties with altered expression of cell adhesion molecules and cytoskeleton protein (epithelial marker: E-cadherin), transforming to mesenchymal cells with dynamic motility and invasive properties (mesenchymal protein: N-cadherin) (18). During the transition, Snail serves as an important EMT transcription factor to induce EMT, which downregulates the expression of epithelial markers and upregulates the expression of mesenchymal markers (19). Thus, we determined the effect of USP1 on the expression of EMT-related protein. Intriguingly, ablation of USP1 increased expression of epithelial marker E-cadherin and decreased expression of mesenchymal markers, N-cadherin and Snail, indicating that USP1 is involved in EMT process (Fig. 2*H*).

### USP1 promotes autophagic flux

**Figure 3:**
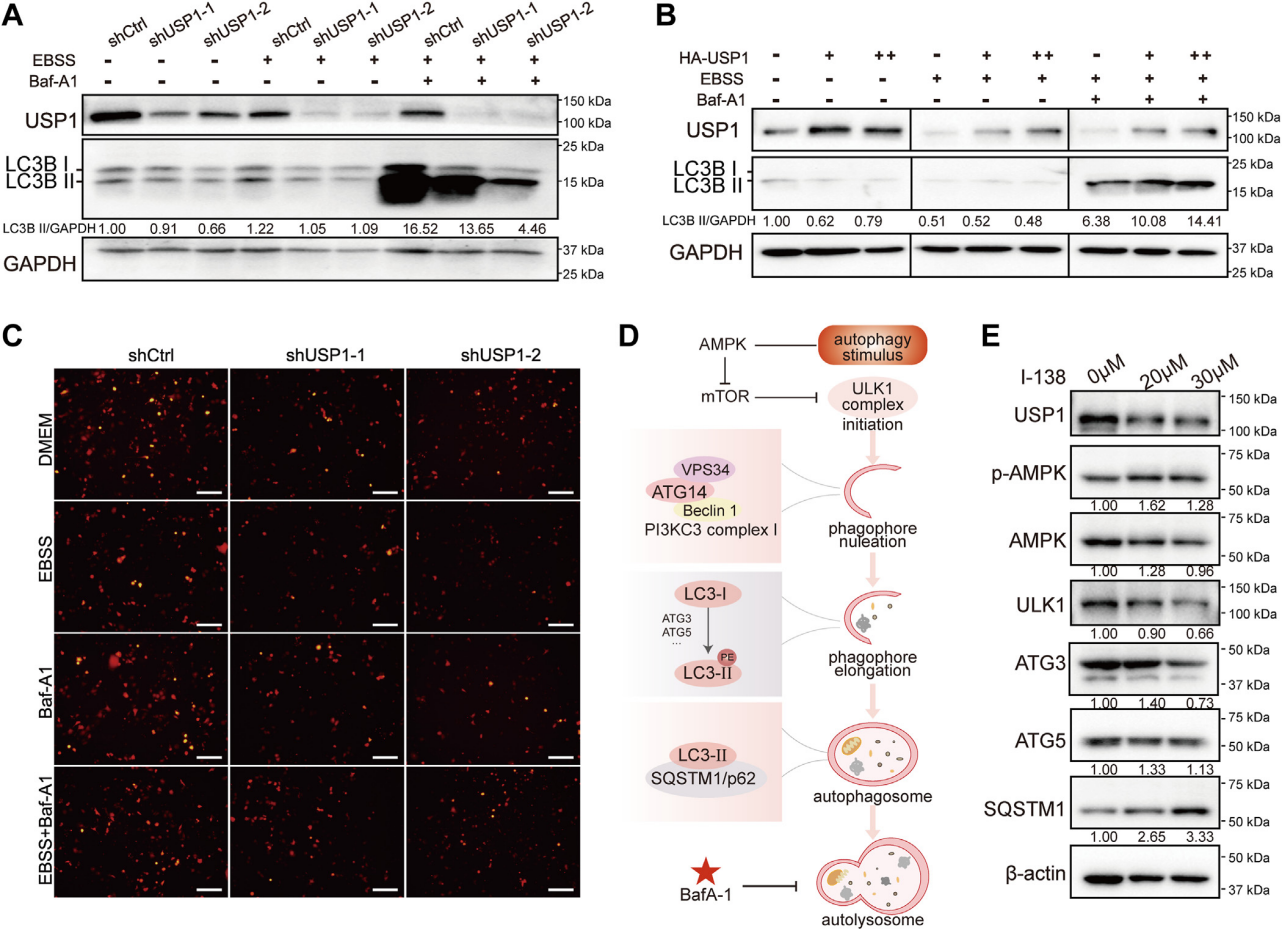
**USP1 promotes autophagy flux.***A* and *B*, the effect of USP1 knockdown or overexpression on autophagy marker LC3B Ⅱ expression with different conditions (normal medium, EBSS induced and Baf-A1). *C*, the effect of USP1 knockdown on LC3B Ⅱ fluorescence using mCherry-LC3 plasmids under different conditions (normal DMEM, EBSS induced, and Baf-A1). Bar represents 200 μm. *D*, brief diagram of autophagy process. *E*, the effect of USP1 inhibition on autophagy-related proteins. Baf-A1, bafilomycin A; DMEM, Dulbecco's modified Eagle's medium; EBSS, Earle's balanced salt solution; USP1, ubiquitin-specific peptidase 1.

Autophagy, a conserved process to recycle organelles and cellular components, is upregulated in PDAC and is implicated in resistance to both cytotoxic chemotherapy and target therapy (7). We have found that USP1 enhanced malignant features of PDAC cells, including cell proliferation and migration. Accordingly, we focus on the role of USP1 in modulating autophagy. During autophagy, cytosolic LC3 (LC3-I) incorporates into autophagosomal membranes and forms LC3-phosphatidylethanolamine conjugate (LC3-II). When autophagosomes fuse with lysosomes to form autolysosomes, LC3-II protein is degraded (20). Thus, monitoring LC3-II protein levels becomes a reliable method for investigating autophagy (20, 21). We detected LC3B Ⅱ (microtubule-associated protein 1 light chain 3 II) protein by Western blotting. The results showed that USP1 knockdown decreased LC3B Ⅱ expression at the basal level and under Earle's balanced salt solution (EBSS)–induced autophagy condition. More importantly, depletion of USP1 further inhibited LC3B Ⅱ expression after treated with bafilomycin A1 (Baf-A1), which raises lysosomal pH to block autophagic flux (Fig. 3*A*). Similarly, overexpression of USP1 induced LC3B Ⅱ expression under EBSS-induced autophagy and Baf-A1 addition (Fig. 3*B*). Then, we transfected mCherry-LC3B plasmid into PANC-1 cells to further explore autophagic flux. The results showed that USP1 knockdown inhibited the fluorescent number of LC3B, suggesting that knockdown of USP1 inhibits cell autophagy (Fig. 3*C*). After autophagy induced by EBSS or inhibited by Baf-A1, the fluorescent number of LC3B in the USP1 knockdown group was also significantly decreased (Fig. 3*C*). SQSTM1/p62 directly binds with LC3 and is degraded by autophagy (20). We found that USP1 inhibition increased the protein expression of SQSTM1/p62 (Fig. 3*E*), indicating that USP1 promotes cellular autophagy.

### USP1 interacts with autophagy-related protein ATG14

The autophagy process is tightly regulated by multiple autophagy-related proteins as shown in Figure 3*D*. Under starvation, the energy sensor adenosine monophosphate–activated protein kinase and mammalian target of rapamycin kinase modulate autophagy-related proteins to involve in the autophagosome formation, including phagosome nucleation, phagosome elongation, expansion, and closure. Then, mature autophagosomes fuse with the lysosome to form autolysosome, and the sequestered contents are subsequently degraded by lysosomal protease (22, 23, 24). We detected the alteration of autophagy-related proteins after inhibition of USP1 by Western blotting. We found that USP1 inhibition increased phosphorylated adenosine monophosphate–activated protein kinase levels and reduced ULK-1 protein levels (Fig. 3*E*). ULK-1 is a component of preinitiation complex, which activates class III PI3K (PIK3C3) complex I to induce autophagy initiation (23). It is reported that USP1 targets ULK1 and regulates canonical autophagy in breast cancer (25), which is consistent with our result in PDAC cells (Fig. 3*E*). Baf-A1 blocks fusion of autophagosomes with lysosomes and autolysosome formation. Since USP1 knockdown inhibited autophagy marker LC3B expression after addition of Baf-A1, we hypothesized that USP1 may induce autophagy prior to autolysosome formation. During autophagosome formation, LC3-I is modified by ubiquitin-like systems including ATG7 and ATG3, and processed by ATG5–ATG12–ATG16, coupled with phosphatidylethanolamine to form LC3-II and localize to the autophagosome membrane (26). We found that USP1 inhibition did not alter the expression of ATG3 and ATG5 (Fig. 3*E*). We focused on preceding step of phagophore elongation, phagophore nucleation process, which is regulated by PIK3C3 complex. The human PI3KC3 complex includes two distinct complexes (C1 and C2). PIK3C3–C1 is essential for autophagosome nucleation, including PIK3C3/VPS34, ATG14, and BECN1. PIK3C3–C2 modulates vesicle trafficking and autophagosome formation, including PIK3C3/VPS34, BECN1, and UVRAG (UV radiation resistance–associated gene) (27, 28).

**Figure 4:**
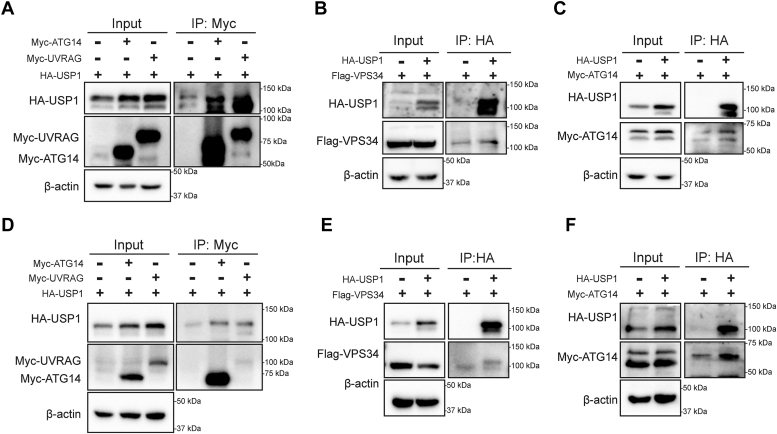
**USP1 interacts with ATG14 protein.***A*–*C*, the indicated plasmids were transfected into HEK-293T cells. The immunoprecipitation experiment was performed with anti-Myc or anti-HA magnetic beads, and cell lysate was detected to investigate the interaction between ATG14, UVRAG, and VPS34 with USP1 in HEK-293T cells. *D*–*F*, the immunoprecipitation experiment was performed to detect the interaction between ATG14, UVRAG with USP1 in PANC-1 cells. ATG14, autophagy-related gene 14; HEK-293T, human embryonic kidney 293T cell line; USP1, ubiquitin-specific peptidase 1; UVRAG, UV radiation resistance–associated gene.

By analysis of The Cancer Genome Atlas database, the mRNA level of *USP1* was positively correlated with *ATG14*, *UVRAG*, and *PIK3C3* (VPS34) (Fig. S2, *A*–*C*). Then, we first detected whether USP1 may interact with ATG14, UVRAG, or VPS34 protein. By using anti-Myc tag magnetic beads, Myc-ATG14 and Myc-UVRAG proteins were successfully enriched, and then USP1 protein was also coprecipitated, indicating that exogenous ATG14 and UVRAG bound with USP1 in human embryonic kidney 293T (HEK-293T) cells (Fig. 4*A*). Interestingly, after immunoprecipitation (IP) of USP1 protein, ATG14 was also coprecipitated, whereas VPS34 and UVRAG were not coprecipitated in HEK-293T cells (Figs. 4, *B* and *C* and S3*A*). Furthermore, we observed that USP1 interacted with ATG14 and not with VPS34 and UVRAG in PANC-1 cells (Figs. 4, *D*–*F* and S3*B*). Besides, VPS34 and Beclin-1 proteins were examined by Western blotting after USP1 knockdown or overexpression, and no significant changes were found (Fig. S4, *A*–*C*). Thus, USP1 may bind with ATG14 to modulate autophagy initiation.

### USP1 deubiquitinates and stabilizes ATG14 protein

ATG14 not only plays a crucial role in autophagy initiation but also participates in tumor growth and drug resistance in colorectal cancer, ovarian cancer, hepatocellular cancer, and pancreatic cancer (29, 30, 31, 32). We demonstrated that ATG14 expression was elevated in tumor tissues compared with normal tissues, and elevated ATG14 expression was correlated with poor prognosis (Fig. S4, *A* and *B*), suggesting that ATG14 may be involved in tumor progression in PDAC.

**Figure 5:**
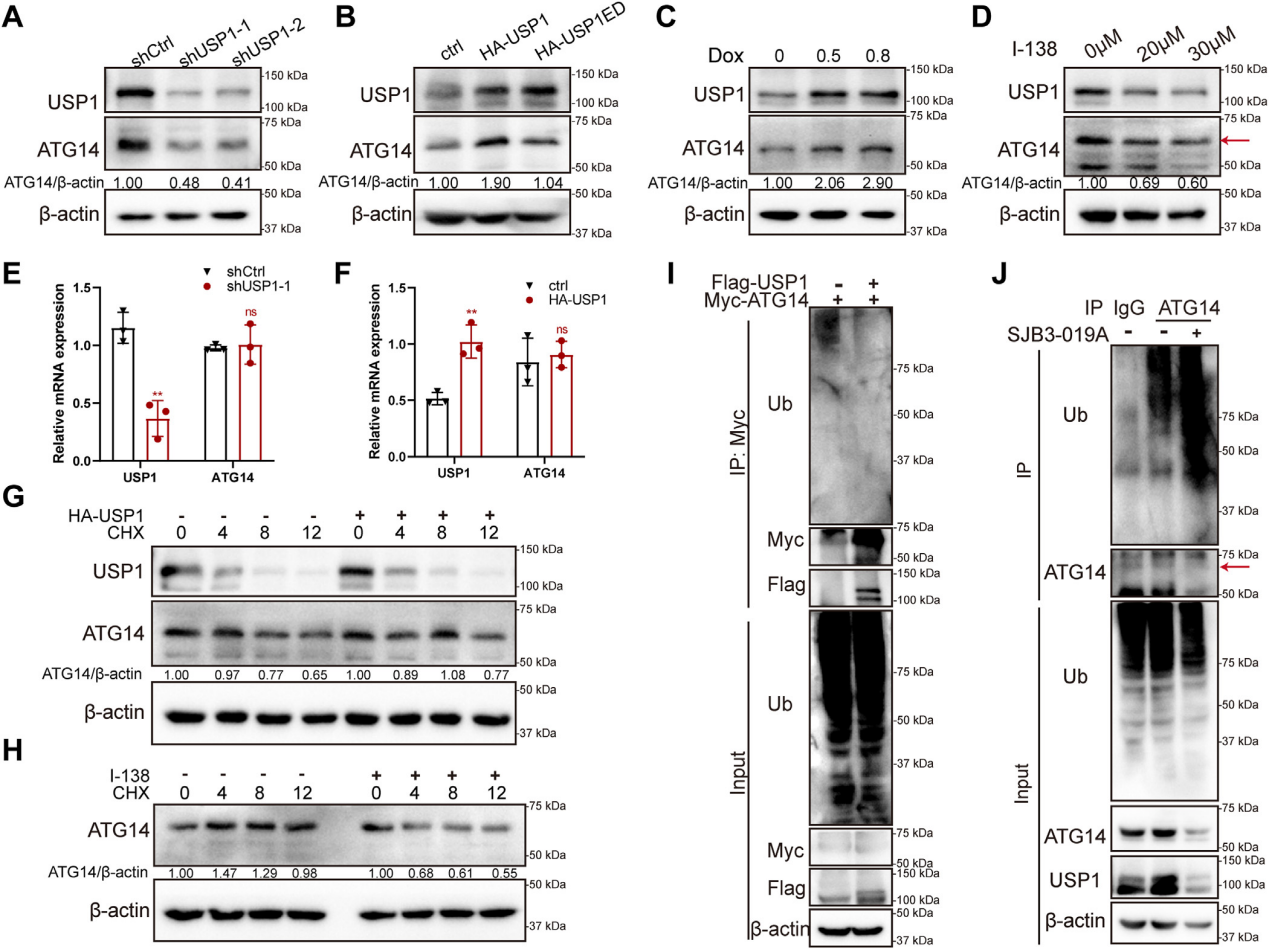
**USP1 deubiquitinates and stabilizes ATG14.***A*, the effect of USP1 knockdown on ATG14 protein levels. *B*, the effect of WT and enzyme-dead mutant C90S of USP1 on the protein levels of ATG14. *C*, the effect of doxycycline-inducible USP1 overexpression on protein levels of ATG14 (doxycycline concentration 0.5, 0.8, and 1.0 μg/ml). *D*, the effect of USP1 inhibitor I-138 on ATG14 protein levels. *E* and *F*, the effect of USP1 overexpression or knockdown on mRNA level of ATG14 by quantitative RT–PCR. Data are shown as mean ± SD of three biological replicates, compared with shCtrl/Ctrl, unpaired *t* test. ∗*p* < 0.05, ∗∗*p* < 0.01, ns, no significance. *G* and *H*, CHX assay was used to detect the effect of USP1 overexpression and pharmacological inhibition (I-138) on protein stability of ATG14 at 4, 8, and 12 h. *I*, ubiquitin assay was used to detect the effect of USP1 overexpression on ATG14-binding ubiquitin. *J*, the effect of USP1 inhibitor SJB3-019A on ATG14 ubiquitin levels. The *red arrow* indicates the target bands. ATG14, autophagy-related gene 14; CHX, cycloheximide; USP1, ubiquitin-specific peptidase 1.

The protein ubiquitination has been revealed as an important mechanism in regulating autophagy-related proteins (33). The E3 ubiquitin ligase MARCH7 ubiquitinates ATG14 to inhibit autophagy (34). However, the exact DUBs for modulating ATG14 are still unknown. We have confirmed that USP1 interacted with ATG14, thus we supposed that ATG14 may be a direct deubiquitination substrate of USP1. We detected whether USP1 regulated ATG14 protein levels. As expected, ablation of USP1 diminished the protein levels of ATG14 (Fig. 5*A*). Similarly, USP1 inhibition also decreased ATG14 protein using a USP1 selective inhibitor I-138 (Fig. 5*D*). Conversely, transient overexpression of USP1 increased ATG14 protein levels (Fig. 5*B*). We also observed that USP1 promoted ATG14 protein in doxycycline-inducible USP1-overexpression cells (Fig. 5*C*). However, overexpression of USP1 deubiquitinase-dead mutant (USP1 C90S) did not alter ATG14 protein levels, indicating that USP1 promotes ATG14 protein levels depending on its deubiquitination activity (Fig. 5*B*). In addition, USP1 did not modulate the mRNA levels of ATG14 (Fig. 5, *E* and *F*). Furthermore, cycloheximide (CHX) assay showed that USP1 overexpression decreased protein degradation of ATG14 and stabilized ATG14 protein (Fig. 5*G*), whereas USP1 inhibitor I-138 treatment resulted in decreased protein stability of ATG14 (Fig. 5*H*). The protein ubiquitin assay demonstrated that USP1 overexpression decreased the ubiquitin level of ATG14 (Fig. 5*I*). Intriguingly, USP1 inhibition by SJB3-019A increased the ubiquitin level of ATG14 (Fig. 5*J*), indicating that USP1 deubiquitinates and stabilizes ATG14 protein.

### USP1 promotes pancreatic tumor growth *in vivo*

**Figure 6:**
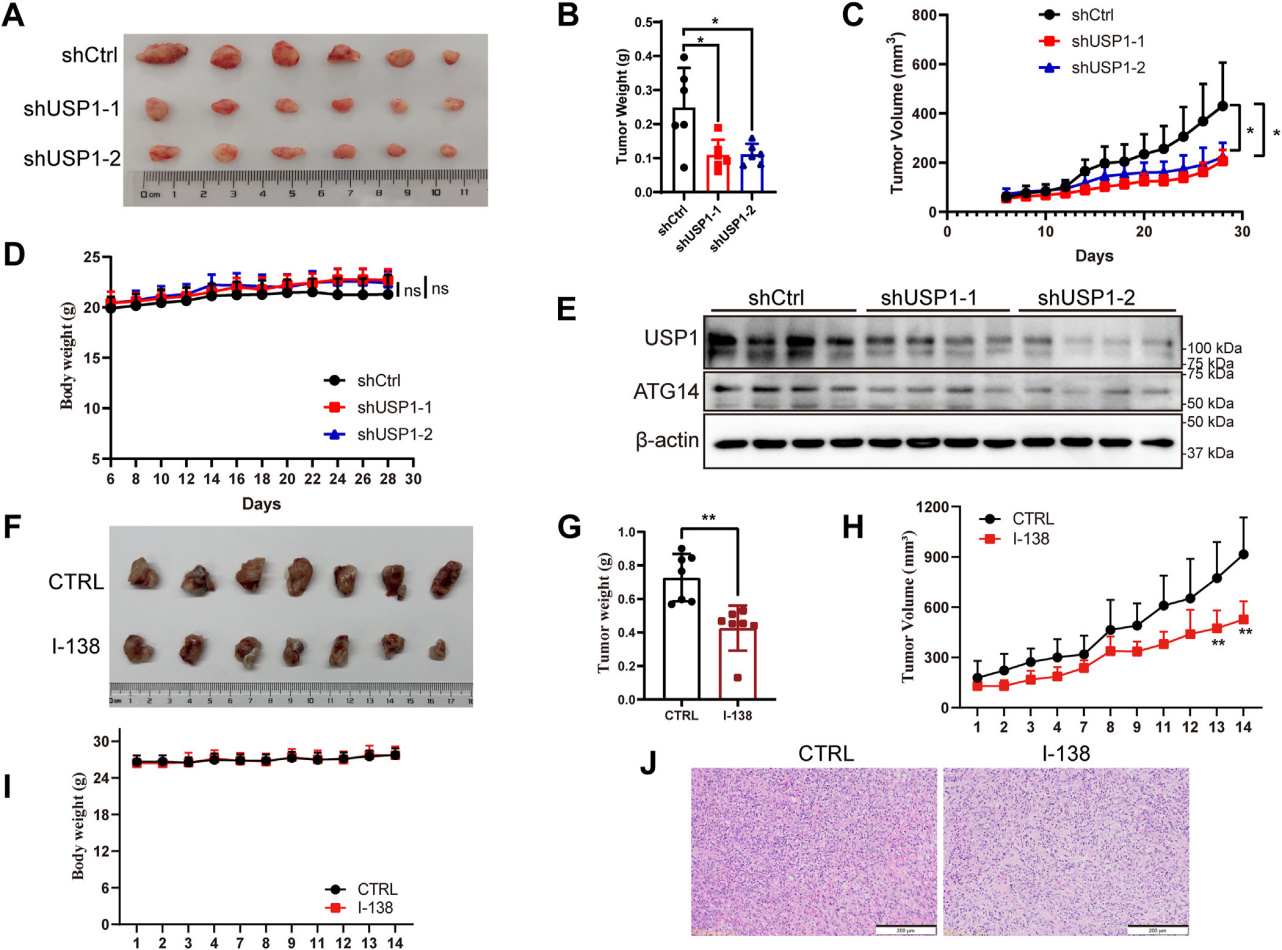
**USP1 promotes tumor growth *in vivo*.***A*–*D*, the PANC-1 xenograft model was established in BALB/c nude mice (n = 6) using control shRNA (shCtrl) and USP1 shRNA (shUSP1-1, shUSP1-2). Tumor images (*A*), tumor weight (*B*), tumor volume (*C*), and the curves of body weight (*D*) were shown from shCtrl and shUSP1 tumors. Data are shown as mean ± SD, compared with shCtrl, unpaired *t* test compared with Ctrl, ∗*p* < 0.05, ns, no significance. *E*, the expression of USP1 and ATG14 in shCtrl and shUSP1 tumor tissues. *F*–*I*, the murine KPC1199 xenograft model was established in C57/BL6 mice (n = 7). The mice were treated with vehicle control (CTRL) and USP1 inhibitor I-138 by oral gavage after tumor volume reached 150 mm³. Images of tumor tissues (*F*), tumor weight (*G*), tumor volume (*H*), and the curves of body weight (*I*) were shown from CTRL and I-138 administration groups. *J*, representative images of HE staining of tumor tissues in CTRL and I-138 administration groups. Compared with CTRL, unpaired *t* test, ∗*p* < 0.05, ∗∗*p* < 0.01, and ∗∗∗*p* < 0.001. ATG14, autophagy-related gene 14; USP1, ubiquitin-specific peptidase 1.

To investigate the role of USP1 in tumor growth *in vivo*, the tumor xenograft model was established by injecting scramble shRNA (shCtrl) and shUSP1 PANC-1 cells into BALB/c nude mice. Interestingly, depletion of USP1 remarkably decreased tumor weight and tumor volume (Fig. 6, *A*–*C*), which did not affect the body weight (Fig. 6*D*). Moreover, ablation of USP1 diminished the expression of ATG14 in tumor tissues (Fig. 6*E*), which suggested that USP1 promotes tumor growth *via* upregulating ATG14 expression. Then, we evaluated antitumor effect of USP1 inhibitor I-138 *in vivo*, which is a novel USP1–USP1-associated factor 1 inhibitor, structurally related to ML323 (35). The murine KPC1199 cells were injected subcutaneously into C57/BL6 mice, and the I-138 inhibitor was administered when the tumor grew to 150 mm^3^. After 2 weeks of administration, I-138 significantly shrunk tumor weight and tumor volume (Fig. 6, *F*–*H*). But I-138 did not influence the body weight (Fig. 6*I*). Taken together, genetic depletion or pharmacologic inhibition of USP1 suppresses pancreatic tumor growth *in vivo*, suggesting that targeting USP1 provides a promising therapeutic strategy in PDAC therapy.

### The pharmacological inhibition of USP1 synergistically enhances the efficiency of cisplatin in PDAC cells

**Figure 7:**
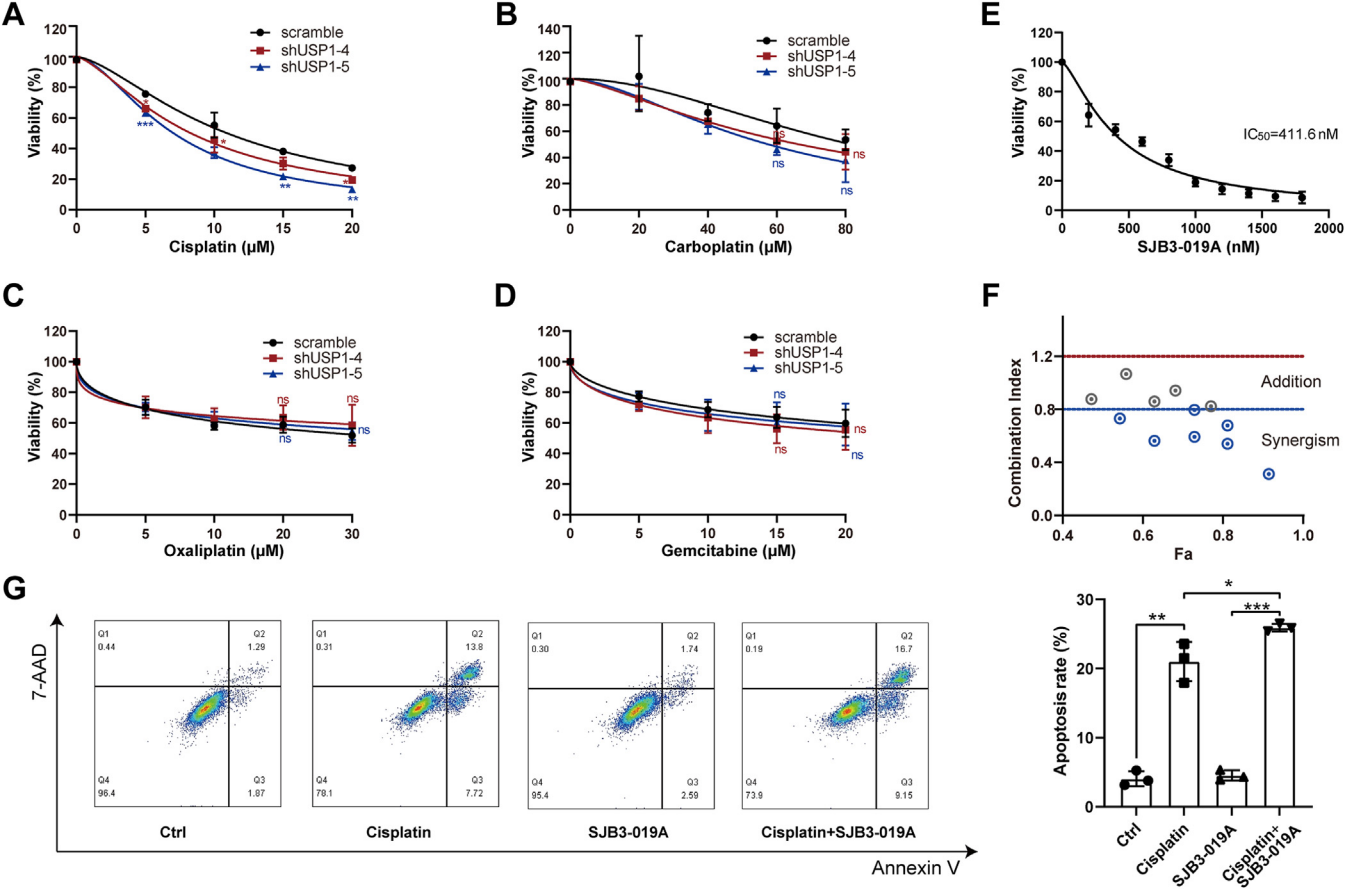
**USP1 inhibition synergistically enhances the anticancer efficiency of cisplatin in PDAC cells.***A*–*D*, cell sensitivity to cisplatin, carboplatin, oxaliplatin, and gemcitabine in control (shCtrl) and shUSP1 cells was determined. Data are shown as mean ± SD from three independent experiments, compared with scramble, two-way ANOVA, ∗*p* < 0.05, ∗∗*p* < 0.01, and ∗∗∗*p* < 0.001. *E*, the effect of USP1 inhibitor SJB3-019A on cell viability by CCK8. *F*, the cell viability of PANC-1 cells was measured by CCK8 with different concentrations of SJB3-019A plus cisplatin, and combination index (CI) was calculated using Compusyn software. Less than 0.8 implied a synergistic effect of drug combination. *G*, the effect of combination of SJB3-019A with cisplatin on cell apoptosis. Data are shown as mean ± SD from three independent experiments, one-way ANOVA compared with Ctrl/SJB3-019A/cisplatin, ∗*p* < 0.05, ∗∗*p* < 0.01, and ∗∗∗*p* < 0.001. CCK8, Cell Counting Kit-8; PDAC, pancreatic ductal adenocarcinoma; USP1, ubiquitin-specific peptidase 1.

The aforementioned results determined that USP1 is a potential therapeutic target for pancreatic cancer. USP1 plays an important role in DNA damage response (DDR) by deubiquitinating proliferating cell nuclear antigen and Fanconi anemia group D2 protein (35, 36). The study showed that USP1 upregulation confers platinum resistance to ovarian cancer cell, and inhibiting USP1 reverses platinum resistance (37). Therefore, targeting USP1 may enhance the sensitivity of chemotherapy drugs. Then, we determined whether depletion of USP1 sensitized PDAC cells to clinical drugs, including gemcitabine- and platinum-based drugs. The CCK8 assay showed that USP1 knockdown exhibited an increased sensitivity to cisplatin, not carboplatin, oxaliplatin, or gemcitabine (Fig. 7, *A*–*D*). Then, USP1 inhibitor SJB3-019A was used to detect if it synergized with cisplatin in pancreatic cancer treatment. Combination index was less than 0.8 at multiple dose combinations, suggesting a synergistic effect between SJB3-019A and cisplatin (Fig. 7, *E* and *F*). More importantly, combination of SJB3-019A with cisplatin significantly accelerated cell apoptosis (Fig. 7*G*), which provides a potential strategy for pancreatic treatment.

## Discussion

PDAC is a highly malignant and aggressive tumor, and the overall survival rate is quite unsatisfying with merely about 10% at 5 years. The chemotherapy drug resistance and immune-desert tumor microenvironment significantly limited therapeutic outcome in PDAC. Mounting evidence showed that USP1 facilitates cancer progression in numerous cancer types, such as osteosarcoma (38), gastric cancer (39, 40) and prostate cancer (41). Interestingly, Qunying-Lei group found that USP1 modulates metabolic reprogramming to induce the progression of PanIN stages to PDAC (17). USP1 also plays an important role in antiviral immune responses *via* cGAS, TBK1, and pSTAT1 (42, 43, 44). Besides, USP1 promotes Th17-cell differentiation but attenuates Treg-cell differentiation (45). Here, we observed the antitumor effect of depletion or pharmacological inhibition upon USP1 in PDAC in both immunodeficient and immunocompetent xenograft models. The effect may be due to the oncogenic role of USP1 in tumor cells and/or tumor immune escape in tumor-infiltrating lymphocytes, the latter of which is worth further investigation.

In pancreatic cancer, autophagy has been reported to be hyperactivated (46). The possible mechanisms are as follows: (1) microphthalmia/transcription factor E family transcription factors drive expression of several genes in autophagy-lysosome activation process (6); (2) the core autophagy protein ULK1 maintains high levels mediated by dephosphorylations of PTPA/PP2A and ubiquitination of E3 ligase NEDD4L (7, 11, 47). Mounting studies show that gene depletion (*e.g.*, atg5^−/−^, atg7^−/−^, or hmgb1^−/−^) and pharmacological inhibition (*e.g.*, using chloroquine) of autophagy ultimately inhibit the development of pancreatic cancer *in vitro* and *in vivo* (48, 49, 50, 51), indicating increased autophagy as a potential therapeutic target in PDAC. While hydroxychloroquine remains frustrating in PDAC clinical trials. Therefore, targeting upstream modulators of affecting autophagy may provide a reasonable clinical strategy. Here, we investigated the role of USP1 in autophagy and revealed that ablation of USP1 suppressed autophagy in PDAC, which not only provides a new mechanism for autophagy activation in pancreatic cancer but also identifies USP1 as a therapy target for pancreatic cancer. Similarly, Marzia Raimondi *et al.* (25) demonstrated that depletion of USP1 impairs canonical autophagy in breast cancer by targeting ULK1, which is consistent with our results. However, in B-cell lymphoma and hepatocellular carcinoma, USP1 inhibition greatly enhances autophagic activity (52, 53). The apparently opposite roles of USP1 in regulating autophagy may be due to different cellular and molecular contexts or tumor heterogeneity.

ATG14 is necessary for autophagosome biogenesis but also promotes membrane fusion between autophagosomes and endolysosomes (54, 55, 56), indicating a crucial role of ATG14 in various steps of autophagy. It has been reported that E3 ubiquitin ligase MARCH7 and ZBTB16–Cullin3–Roc1 are involved in the ubiquitination regulation of ATG14 (34, 57). We identified USP1 as the first deubiquitinase and modulated ATG14 deubiquitination. K94, K124, K136, K143, K150, K171, K236, K328, and K341 of ATG14 may be potential ubiquitylation sites according to mass spectrometry data on PhosphoSitePlus website (https://www.phosphosite.org/). However, the specific deubiquitination sites on ATG14 by USP1 and the ubiquitin type of lysine need to be determined through experiments in further study. In addition, how USP1 and the specific E3 ligase collaboratively responsible for maintaining ATG14 protein stability in PDAC merits further investigation.

Previous research has demonstrated that autophagy is implicated in resistance to chemotherapy (58). ATG14 is found to protect auditory hair cells from cisplatin cytotoxicity (59). In addition, ATG14 promotes platinum drugs and gemcitabine resistance in colorectal cancer, pancreatic cancer, ovarian cancer, and lung cancer (29, 32, 37, 60). Thus, targeting ATG14 may be a potential strategy for PDAC treatment. Unfortunately, the selective ATG14 inhibitors are currently absent. We have found that USP1 acted as an upstream regulator of ATG14 in PDAC. Then, we evaluated depletion or pharmacological inhibition of USP1 on platinum sensitivity. As a DUB, USP1 regulates several factors of DDR, especially proliferating cell nuclear antigen and Fanconi anemia group D2 protein, which plays an important role in translesion synthesis and Fanconi anemia pathway (35, 36, 61). The possible mechanisms of enhanced sensitivity to cisplatin upon USP1 inhibition may be due to (1) the role of ATG14 dependent on autophagy modulation and (2) crucial roles of USP1 in DDR regardless of autophagy.

In summary, this study provides critical insights into the mechanism of USP1 in PDAC aggression and autophagy. Our finding reveals a positive association between increased USP1 expression and a poor outcome in PDAC patients. USP1 deubiquitinates and stabilizes ATG14, thus remarkably promoting malignant features, such as cell proliferation, migration, autophagy, and tumor growth in PDAC. Targeting USP1 significantly inhibits the tumorigenic ability and exhibits a synergistic antitumor effect in combination with cisplatin in preclinical models. Hence, our study identifies USP1 as a promising target, and targeting USP1 may offer a therapeutic avenue for PDAC treatment.

## Experimental procedures

### Cell culture and reagents

The PDAC cell lines PANC-1, BxPC3, ASPC1, CFPAC-1 and embryonic kidney HEK-293T cells were obtained from Cell Bank of the Chinese Academy of Sciences. HPNE (normal human pancreatic duct epithelial cell) was purchased from MeisenCTCC. The murine pancreatic cancer cell line KPC1199 was kindly provided by Professor Jing Xue from Shanghai Jiao Tong University. PANC-1, HPNE, KPC1199, and HEK-293T were cultured in Dulbecco's modified Eagle's medium (Gibco), and BxPC3, ASPC1, and CFPAC-1 were cultured in RPMI1640 medium (Gibco). All cell lines were supplemented with 10% fetal bovine serum (ExCell Bio) and penicillin–streptomycin solution (Beyotime) in a humidified atmosphere of 5% CO_2_ at 37 °C. The compounds cisplatin, carboplatin, oxaliplatin, gemcitabine, and USP1 inhibitor I-138 were obtained from Selleck Chemicals, and another USP1 inhibitor SJB3-019A was obtained from MedChemExpress.

### Plasmid transfection and virus infection

FLAG-tagged USP1 and HA-tagged USP1 (WT and C90S mutant) were cloned into the pcDNA3 vector. mCherry-LC3, Myc-ATG14, FLAG-VPS34, and Myc-UVRAG were obtained from Addgene. The RNA interference sequences of scramble (shCtrl) and USP1 (shUSP1) were listed as follows: shCtrl: CCTAAGGTTAAGTCGCCCTCG; shUSP1-1: GCAGATTATGAGCTATACAAC; and shUSP1-2: GACTGAATAATCTCGGCAATA. The shRNA sequences were cloned into pLKO-EGFP-puro vector (MiaoLing Biology). The shCtrl and shUSP1 plasmids were cotransfected with pMD2.G and psPAX2 into HEK-293T cells with polyethyleneimine (Polysciences) transfection reagent. Fresh medium was replaced after transfection for 6 h and 48 h later, cell supernatants were collected, and the virus was purified using PEG8000 (Millipore), then stored at −80 °C. Pancreatic cancer cells were infected with shCtrl and shUSP1 virus containing 8 μg/ml polybrene (Millipore), and the knockdown efficiency was detected by quantitative RT–PCR and Western blot.

### CCK8 assay

For cell proliferation assay, 2500 cells were seeded into 96-well plate and 10% CCK8 (Selleck) was added into cell medium, and the absorbance at 450 nm was measured daily for successive 4 to 8 days.

For drug sensitivity, cisplatin (1, 10, 15, and 20 μM), carboplatin (20, 40 ,60, and 80 μM), oxaliplatin (5, 10, 20, and 30 μM), gemcitabine (5, 10, 15, and 20 μM), and SJB3-019A (0.2, 0.4, 0.6, 0.8, 1.0, 1.2, 1.4, 1.6, and 1.8 μM) were separately added into cell medium, and after 48 h–72 h treatment, cell viaility was measured by CCK8.

For drug combinations, the USP1 inhibitor SJB3-019A (0.4, 0.5, and 0.6 μM) and cisplatin (5, 10, 15 and 20 μM) were separately or together added into the cells. After 48 h treatment, cell viability was measured by CCK8. Compusyn software (ComboSyn, Inc.) was used to calculate combination index. Less than 0.8 implies a synergistic effect of drug combination, whereas more than 1.2 implies antagonistic effect.

### EdU and colony formation assay

EdU staining was performed according to the manufacturer’s instructions (Beyotime). In brief, 100 μM EdU solution was added into cell medium for 2 h, then fixed with paraformaldehyde, and permeabilized with 0.3% Triton X-100. Cells were incubated with EdU and Hoechst 33342, and then fluorescent microscopy was used for imaging.

For colony formation assay, cells were seeded into 12-well plates and cultured about 10 days. The medium was refreshed every 3 days. The cells were washed twice with PBS, fixed with 4% paraformaldehyde, and stained with 0.1% crystal violet.

### Transwell assay

For migration assay, cells were suspended in fetal bovine serum–free medium and seeded in the upper compartment, and the lower compartment of the chamber was added with 500 μl complete medium. After 24 h, cells were fixed with paraformaldehyde and stained with 0.1% crystal violet.

### Cell apoptosis assay

The Annexin V–7-aminoactinomycin apoptosis assay was performed according to the manufacturer’s instructions (Peprotech). After compound treatment, cells were digested with EDTA-free trypsin, collected and washed by PBS, and then resuspended with Annexin V Binding buffer. The Annexin V and 7-aminoactinomycin were added and incubated away from light, following detection by flow cytometry.

### Quantitative real-time PCR

The total RNA was isolated using RNA isolation kit (Fastagen). The reverse transcription was performed with ReverTra Ace RT Master Mix with gDNA Remover (TOYOBO). The quantitative RT–PCR was conducted using The Fast SYBR Green Master Mix (Selleck) according to the manufacturer's instructions. GAPDH was employed as the control, and the relative expression of target genes was calculated by the 2^-ΔΔCT^ method. The primers are listed in Table S1.

### Western blot and coimmmunoprecipitation

Cells were lysed in radioimmunoprecipitation assay buffer (Beyotime) containing protease and phosphatase inhibitor (Selleck) at 4 °C. Then, protein samples were separated on an SDS-PAGE and transferred onto polyvinylidene difluoride membrane (Millipore). After blocked in 5% skim milk (Beyotime) for 2 h, the membranes were incubated in primary antibodies at 4 °C overnight. After that, the membranes were washed with Tris-buffered saline with Tween-20 and probed with horseradish peroxidase–conjugated secondary antibodies (CST; 7074S or 7076S) at room temperature for 2 h. Subsequently, the bands were visualized by enhanced chemiluminescence kit (Proteintech). Integrated densities of immunoblots were quantified by ImageJ software (National Institutes of Health), and relative expression was calculated as integrated density ratio of target protein/internal loading control protein. As for co-IP, cells were lysed in IP lysis buffer (Beyotime) and incubated with anti-HA/Myc magnetic beads (Selleck) at 4°C overnight. Immunoprecipitated proteins were eluted and were then detected by Western blotting. The primary antibodies used are listed in Table S2.

### CHX and ubiquitination assay

For CHX assay, 100 μg/ml CHX was added into PANC-1 cells every 4 h. Twelve hours later, cells were collected and lysed in radioimmunoprecipitation assay buffer containing protease and phosphatase inhibitors. After denaturation, protein samples were detected by Western blot.

For ubiquitination assay, Myc-ATG14 and FLAG-USP1 were transfected into HEK-293T cells, and cells were treated with 50 μM MG132 before protein extraction. Cells were lysed in IP lysis buffer (Beyotime) containing protease inhibitor and incubated with Anti-Myc magnetic beads (Selleck) at 4°C overnight. Immunoprecipitated proteins were eluted and detected with antiubiquitin antibody by Western blot. As for endogenous ATG14 ubiquitin level, the USP1 inhibitor SJB3-019A (MCE) and MG132 were used to treat cells. Cell lysis was incubated with Protein A/G beads, which prebound with IgG or ATG14 antibody, and immunoprecipitated proteins were detected with antiubiquitin antibody by Western blot.

### Mouse xenograft model

All animal experiments were approved by the Animal Care and Use Committee for Shandong university. Male BALB/c nude mice were purchased from Vital River Laboratory Animal Technology and maintained at The Model Animal Research Center in Shandong University. For the PANC-1 xenograft model, 1 × 10^7^ cells were implanted subcutaneously into the right flanks of male nude BALB/c mice. Tumors were measured by calipers, and body weights were recorded during the period. After 30 days, BALB/c nude mice were sacrificed, and tumor tissues were harvested, photographed, and weighed.

Male C57/BL6 mice were purchased from Vital River Laboratory Animal Technology. KPC1199 cells at a concentration of 1 × 10^6^ were injected into right subcutaneous tissue. After tumor volume reached 150 mm^3^, mice were randomly divided into two groups and treated with vehicle control (ctrl) and USP1 inhibitor I-138 by oral gavage six times every week, respectively. I-138 was administered at 50 mg/kg in 5% dimethyl sulfoxide (Solarbio) + 40% PEG300 (MCE) + 5% Tween-80 (MCE) + 50% H_2_O. Tumor volume was measured and calculated as (length × width × width)/2. After 14 days, mice were sacrificed, and the tumors were photographed, weighed, and collected for histological staining.

### HE staining and IHC

For HE staining, tumor tissues of mice were collected and fixed with 4% paraformaldehyde for 48 h. The tissues were dehydrated and cleared with different concentrations of alcohol and xylene and then embedded and sliced with paraffin. The slides were deparaffinized, hydration, immersed in hematoxylin for 10 s, and rinsed by water. After that, the tissues were stained with eosin and then dehydrated and clear. The mounting medium was added, followed by a coverslip.

The tissue microarray was obtained from Outdo Biotech Company, and IHC was performed according to the standard protocol. Briefly, the tissue slice was deparaffinized, rehydrated, and performed antigen retrieval. The slide was incubated with USP1 antibody at 4 °C overnight and secondary antibody for 45 min and then developed with 3,3'-diaminobenzidine staining. After hematoxylin staining, dehydrating and clear coverslips were applied with a mounting medium. The criteria for IHC were as follows: no color (negative) and brown staining (positive). The detached tissues were excluded for analysis.

### Statistical analysis

All experiments were repeated at least three times. Statistical analyses were performed using GraphPad Prism 9.0 (GraphPad Software, Inc). All data are shown as the mean ± SD. Normality of the data was tested using the Shapiro–Wilk normality test. Pairwise comparisons were performed with Brown–Forsythe test and unpaired Student's *t* test (two-tailed) of which SDs not being significantly different. Data with normal distribution and same SD were analyzed by one-way ANOVA with Dunnett’s post-test for multiple comparisons. For CCK8 assay for detecting sensitivity to drugs upon USP1 knockdown, repeated-measures two-way ANOVA was used with Tukey’s multiple comparisons test. Statistical significance was indicated as ∗*p* < 0.05, ∗∗*p* < 0.01, and ∗∗∗*p* < 0.001.

## Data availability

The data that support the findings of this study are available on request from the corresponding author.

## Supporting information

This article contains supporting information.

## Conflict of interest

The authors declare that they have no conflicts of interest with the contents of this article.

## Figures and Tables

**renamed_b2657:**
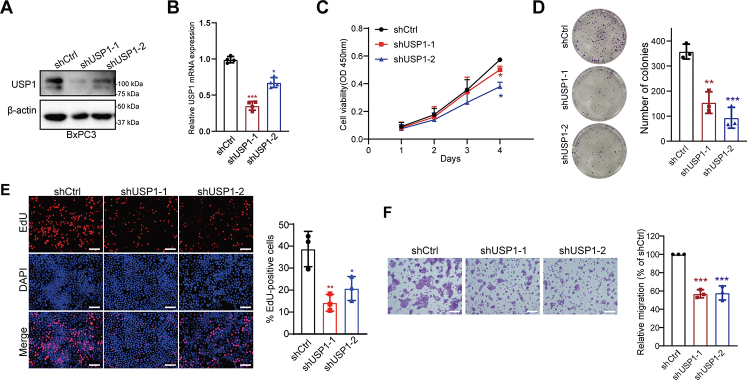


**renamed_50a06:**
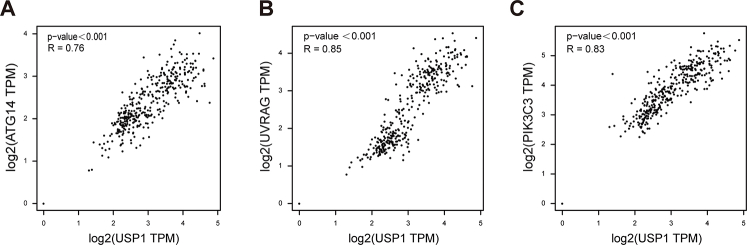


**renamed_35c97:**
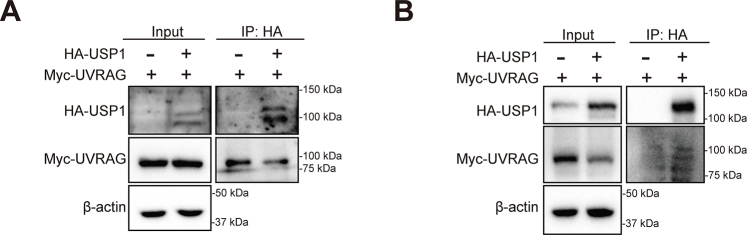


**renamed_c9a77:**
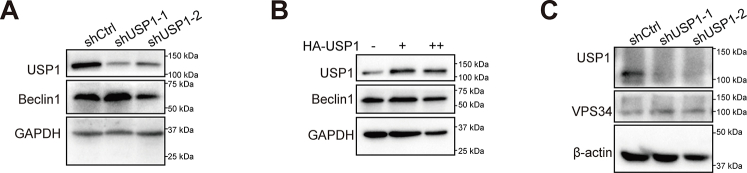


**renamed_ab581:**