# Supplementary material for: USP1 promotes pancreatic cancer progression and autophagy by deubiquitinating ATG14
Source: J Biol Chem. 2025 Jan 13;301(3):108190. doi: 10.1016/j.jbc.2025.108190 (PMC11871461; doi:10.1016/j.jbc.2025.108190)
Supplement: Supporting text [file mmc1.docx]

**USP1 promotes pancreatic cancer progression and autophagy by deubiquitinating ATG14**

# Supplementary Figure legends

**Supplementary Figure 1. USP1 promotes cell proliferation and migration in BxPC3 cells.** (A-B) Detection of USP1 knockdown efficiency at protein and mRNA level in shUSP1 BxPC3 cells. (C) Cell proliferation ability of shCtrl and USP1 knockdown cell lines measured by CCK8. (D-E) Representative images of colony formation and EdU staining in Ctrl and USP1 knockdown cell lines. Bar=200μm. (F) Effect of USP1 on cell migration ability in BxPC3 cells. Bar=200μm. Data are shown as mean ± S.D. from three independent experiments. Compared with shCtrl, one-way ANOVA. *p < 0.05, **p < 0.01, ***p < 0.001.

**Supplementary Figure 2. USP1 mRNA is positively correlated with PIK3C3, ATG14 and UVRAG.** (A-C) Correlation between USP1 with ATG14, UVRAG, PIK3C3 according to GEPIA database.

**Supplementary Figure 3. USP1 doesn’t interact with UVRAG.** (A) Immunoprecipitation experiment to detect the interaction between UVRAG with USP1 in HEK-293T cells. (B) Immunoprecipitation experiment to detect the interaction between UVRAG with USP1 in PANC-1 cells.

**Supplementary Figure 4. USP1 doesn’t regulate the expression of Beclin1 and VPS34.** (A-B) the effect of USP1 knockdown and overexpression on expression of Beclin1 in PDAC cells. USP1 and GAPDH in Figure S4B were reused from the same image as the first panel in Figure 3B. (C) Effect of USP1 knockdown on protein expression of VPS34 in PDAC cells.

**Supplementary Figure 5. High ATG14 expression is associated with poor prognosis in pancreatic cancer.** (A) ATG14 protein expression in normal pancreatic tissues and PDAC tissues (compared with normal tissues, ***p < 0.001). (B) Correlation between ATG14 expression and disease-free survival (*p < 0.05).
