# Supplementary material for: USP1 promotes pancreatic cancer progression and autophagy by deubiquitinating ATG14
Source: J Biol Chem. 2025 Jan 13;301(3):108190. doi: 10.1016/j.jbc.2025.108190 (PMC11871461; doi:10.1016/j.jbc.2025.108190)
Supplement: Supporting table [file mmc2.docx]

# Supporting Information

# USP1 promotes pancreatic cancer progression and autophagy by deubiquitinating ATG14

Leilei Li^a^, Zhili Fan^a^, Mengfei Liu^a^, Hao Dong^a^, Jing Li^a^, Yu Li^a^, Zan Song^a^, Ying Liu^a^, Zhicheng Zhang^a^, Xinyu Gu^a^, Tao Zhang^a,b,*^

^a^ Institute of Immunopharmaceutical Sciences, NMPA Key Laboratory for Technology Research and Evaluation of Drug Products, Key Laboratory of Chemical Biology, School of Pharmaceutical Sciences, Cheeloo College of Medicine, Shandong University, Jinan, Shandong, China

^b^ State Key Laboratory for Chemistry and Molecular Engineering of Medicinal Resources, Guangxi Normal University, Guilin, Guangxi, China

^*^ Corresponding author. E-mail address: [zhangtao@sdu.edu.cn](mailto:zhangtao@sdu.edu.cn)

# Supplementary Tables

| **Gene** | **species** | **Forward Primer (5’-3’)** | **Reverse Primer (5’-3’)** |
| --- | --- | --- | --- |
| *GAPDH* | Human | GGAGCGAGATCCCTCCAAAAT | GGCTGTTGTCATACTTCTCATGG |
| *USP1* | Human | CCATCTCAGGGTCTTCATTTCTG | GCGTTGCTTGGAATGTGAAAG |
| *ATG14* | Human | TGTACCTGGTCAGTCCAAGCTC | CAGGTCGGTTTCTTCATCGCTG |

## Table S1. Primer sequences of qRT-PCR

## Table S2. List of western blotting and co-IP antibodies used.

| **Antibody** | **species** | **Supplier** | **Catalog number** | **Dilution** |
| --- | --- | --- | --- | --- |
| USP1 | Rabbit | Proteintech | 14346-1-AP | 1:1000 |
| LC3B | Rabbit | Cell Signaling Technology | 3868S | 1:4000 |
| ATG14 | Rabbit | ABclonal | A7526 | 1:1000 |
| VPS34 | Rabbit | ABclonal | A12483 | 1:1000 |
| UVRAG | Rabbit | ABclonal | A8462 | 1:1000 |
| HA | Rabbit | Cell Signaling Technology | 3724S | 1:2000 |
| Myc | Rabbit | Cell Signaling Technology | 2278S | 1:2000 |
| Flag | Rabbit | Cell Signaling Technology | 14793S | 1:4000 |
| E-cadherin | Rabbit | Proteintech | 20874-1-AP | 1:1000 |
| N-cadherin | Rabbit | Proteintech | 22018-1-AP | 1:1000 |
| Snail (C15D3) | Rabbit | Cell Signaling Technology | 3879S | 1:1000 |
| GAPDH | Mouse | Proteintech | 60004-1-Ig | 1:5000 |
| β-actin | Rabbit | Abclonal | AC026 | 1:20000 |
| Ubiquitin | Mouse | Santa | sc-8017 | 1:500 |
| ATG3 | Rabbit | Abclonal | A19594 | 1:1000 |
| ULK1 | Rabbit | ABmart | T56902F | 1:1000 |
| ATG5 | Rabbit | ABmart | T55766F | 1:1000 |
| p-AMPK(T172) | Rabbit | Cell Signaling Technology | 2535S | 1:1000 |
| AMPK | Rabbit | Cell Signaling Technology | 2532S | 1:1000 |
| Beclin1 | Rabbit | Abclonal | A7353 | 1:1000 |
| SQSTM1/p62 | Rabbit | Cell Signaling Technology | 5114S | 1:1000 |
